# Human telomeres that carry an integrated copy of human herpesvirus 6 are often short and unstable, facilitating release of the viral genome from the chromosome

**DOI:** 10.1093/nar/gkt840

**Published:** 2013-09-19

**Authors:** Yan Huang, Alberto Hidalgo-Bravo, Enjie Zhang, Victoria E. Cotton, Aaron Mendez-Bermudez, Gunjan Wig, Zahara Medina-Calzada, Rita Neumann, Alec J. Jeffreys, Bruce Winney, James F. Wilson, Duncan A. Clark, Martin J. Dyer, Nicola J. Royle

**Affiliations:** ^1^Department of Genetics, University of Leicester, University Road, Leicester, LE1 7RH, UK, ^2^Department of Oncology, ORCRB, University of Oxford, Old Road Campus, Headington, Oxford, OX3 7DQ, UK, ^3^Centre for Population Health Sciences, College of Medicine and Veterinary Medicine, University of Edinburgh, Teviot Place, Edinburgh, EH8 9AG, Scotland, ^4^Department of Virology, Barts Health NHS Trust, Pathology and Pharmacy Building, 80 Newark St, London E1 2ES, UK and ^5^Department of Cancer Studies and Molecular Medicine, University of Leicester, University Road, Leicester, LE1 7RH, UK

## Abstract

Linear chromosomes are stabilized by telomeres, but the presence of short dysfunctional telomeres triggers cellular senescence in human somatic tissues, thus contributing to ageing. Approximately 1% of the population inherits a chromosomally integrated copy of human herpesvirus 6 (CI-HHV-6), but the consequences of integration for the virus and for the telomere with the insertion are unknown. Here we show that the telomere on the distal end of the integrated virus is frequently the shortest measured in somatic cells but not the germline. The telomere carrying the CI-HHV-6 is also prone to truncations that result in the formation of a short telomere at a novel location within the viral genome. We detected extra-chromosomal circular HHV-6 molecules, some surprisingly comprising the entire viral genome with a single fully reconstituted direct repeat region (DR) with both terminal cleavage and packaging elements (PAC1 and PAC2). Truncated CI-HHV-6 and extra-chromosomal circular molecules are likely reciprocal products that arise through excision of a telomere-loop (t-loop) formed within the CI-HHV-6 genome. In summary, we show that the CI-HHV-6 genome disrupts stability of the associated telomere and this facilitates the release of viral sequences as circular molecules, some of which have the potential to become fully functioning viruses.

## INTRODUCTION

The ends of linear chromosomes are distinguished from double-strand breaks within the genome by specialized nucleoprotein structures known as telomeres. Human telomeres comprise a variable-length double-stranded DNA molecule that is predominantly 5′-(TTAGGG)_n_-3′ (1) but includes, at the start of the repeat array, sequence-variant or degenerate repeats interspersed with TTAGGG repeats (2–4). Each telomere is terminated by an essential single-strand overhang of 50–300 nt (5). The telomeric DNA binds to the six-protein complex, known as Shelterin, via the double-strand binding TRF1 and TRF2 proteins and the single-strand binding protein POT1 (6). Telomeric DNA bound to the Shelterin complex forms looped structures (t-loops) in which the single-strand overhang invades the upstream duplex telomeric DNA forming a D-loop (7). The Shelterin complex on the capped telomere prevents inappropriate repair by non-homologous end-joining or by homologous recombination (HR) (8) and, in conjunction with other protein complexes, it regulates telomerase activity by controlling access to the single-strand overhang. However, telomerase is inactive in differentiated human cells and shows limited activity in stem cells; consequently in most cells, telomeric DNA is eroded as a result of incomplete lagging-strand synthesis and end-processing that restores single-strand overhangs (6). Disturbance of the telomere capping function or telomere length regulation can result in genome instability leading to tumourigenesis, and the presence of a few short telomeres (9,10) induces senescence, which has known roles in ageing.

HHV-6A and B belong to the *Roseolovirus* genus of the β-herpesvirus subfamily, and though closely related, they present diverse biological, epidemiological, pathological and molecular properties. HHV-6B is prevalent in most populations and primary infection usually occurs in early childhood (<2 years) causing an intense fever and rash, whereas infections by HHV-6A tend to be less common (11–13). As for most herpesviruses, HHV-6A and B can remain latent lifelong, with the potential to reactivate that can have severe consequences. For example, HHV-6B reactivation in immunocompromised transplant patients can cause encephalitis and has been linked to bone marrow suppression and to graft failure (14). The HHV-6A and B genomes are double-stranded DNA molecules comprising ∼145 kb of unique sequence encoding U1 to U100 ORFs, flanked by identical left and right direct repeats (DR_L_ and DR_R_), each ∼8 kb (15–17). Each DR is terminated by the packaging and cleavage sequences, PAC1 and 2 (18), and includes two arrays of telomere-like repeats (T1 and T2) and a variety of ORFs (DR1–8). T2 is known to be a short array of pure (TTAGGG)_n_ that varies in length between viral isolates, whereas T1 comprises an array of degenerate telomere-like repeats (19,20).

HHV-6A and B are distinct among herpesviruses, as they can integrate into the human telomeric DNA, probably via HR with the terminal T2 region of DR_R_ (21–25). Once integrated in the germline, the chromosomally integrated virus (CI-HHV-6) can be inherited. Approximately 0.8% of the UK population are CI-HHV-6 carriers (26), but it is not known whether integration is a natural biological form of HHV-6 latency or whether it affects normal telomere function and length regulation. We show that the telomere carrying the integrated virus is often the shortest measured and that the presence of the full-length viral genome disrupts stability of the telomere, resulting in frequent truncations. Moreover, we detected extra-chromosomal circular DNA comprising viral sequences and showed that the viral genome can be released from the telomere, possibly via a t-loop excision mechanism. Altogether, our data are consistent with the proposal that telomeric integration is a form of HHV-6 latency.

## MATERIALS AND METHODS

### Oligonucleotides

Oligonucleotides for amplification of HHV6 sequences were primarily designed based on the GenBank consensus sequences for HHV-6A (U1102 accession no. X83413.1) or HHV-6B (HST accession no. AB021506.1; Z29 accession no. AF157706.1).

### DNA samples and lymphoblastoid cell lines

To identify CI-HHV-6 carriers, we screened the HapMap Phase I (27), the CEPH-HGDP (28) and parental DNA samples from the CEPH family panel (29). Therefore, we screened 1178 samples from unrelated donors in these three panels (duplicated samples between the panels were removed). We also screened 528 samples in the People of the British Isles panel (30) and 2153 samples from the Orkney Complex Disease Study (31). The panel of 92 sperm donors, a gift from Alec J. Jeffreys (32), was also screened for the presence of CI-HHV-6.

Lymphoblastoid cell lines (LCLs) from one CI-HHV-6A (designated 3-10q26.3) and five CI-HHV-6B carriers (designated 1-9q34.3, 2-9q34.3, 4-11p15.5, 5-17p13.3 and 6-17p13.3) reported previously (24) were donated by Duncan Clark. Other LCLs were from Bruce Winney: NWA090 (CI-HHV-6A), BAN519, COR264, CUM082, DER512, NWA008, YOR546 (all CI-HHV-6B); from David Gurwitz CEPH-HGDP00628 (CI-HHV6A); from the cell bank at the Coriell Institute for Medical Research: GM18999 (CI-HHV-6A), GM07022 (CI-HHV-6B), CEPH 1375.02 [also known as GM10863 (CI-HHV-6B)] and from other members of the CEPH 1375 family. All the LCLs were grown in RPMI1640 medium with 10–15% foetal calf serum at 37°C in a humidified incubator with 5% CO_2_ and subcultured using standard methods. To measure the telomere shortening rate in the GM18999 (CI-HHV-6A) and CEPH1375.02 (CI-HHV-6B) cell lines, the cells were cultured over a longer period. The number of population doublings was calculated from cell counts obtained using a haemocytometer, and the percentage of dead cells was determined using Trypan blue staining at each subculturing. Cell pellets were obtained throughout the time course.

### Identification of HHV-6-positive DNA samples

Panels of DNA samples in 96-well format were screened by polymerase chain reaction (PCR) for the presence of U11 or U18 sequences. Standard 10 µl PCRs were prepared containing 5 ng genomic DNA, 0.3 µM of each primer, 0.05 U/µl of Taq polymerase (Kapa Biosystems) and a buffer supplied with the enzyme. Thermal cycling conditions were 96°C for 1 min then 35 cycles of 96°C for 15 s; 62–64°C for 30 s; and 68°C for 1 min). Forward and reverse primers: U11F 5′-TTTTTACATCACGACGCGATC; U11R 5′-GGGACGCGAATCGGAGGAAGC; U18F 5′-CATATCTGATCAACCTTGCGATG; and U18R 5′-ATAACAGCATCGTAAATGCACCC. Identification of the virus type was achieved by amplification with primers DR5F(A): 5′-CGTCGACTTCTCGTTCTTTATGC; DR5R(A): 5′-CACATACCATGAACGGACACAC for HHV-6A and DR7F(B): 5′-AGGCG; DR7R(B): 5′-CCGAATACGTCCAATGTCCT for HHV-6B. Amplicons of the expected sizes were detected by agarose gel electrophoresis.

### Single telomere length analysis

Single telomere length analysis (STELA) was conducted as described (33) using the specialized PCR buffer and other modifications reported previously (34,35). The primer concentrations in the 10 µl STELA reactions were flanking primer 0.3 µM, Telorette2 0.225 µM and Teltail 0.05 µM. The *Taq* polymerase (Kapa Biosystems) was used at 0.04 U/µl and *Pwo* (Genaxxon Bioscience) at 0.025 U/µl. The primers located adjacent to the telomere repeat and used to amplify the double-stranded portion of the 12q, 17 p and XpYp telomeres were 12q-STELA: 5′-CGAAGCAGCATTCTCCTCAG; 17p6: 5′-GGCTGAACTATAGCCTCTGC; XpYpE2: 5′-TTGTCTCAGGGTCCTAGTG. Primers used for STELA from the integrated HHV-6A or B were DR1R: 5′-GAAGAAGATGCGGTTGTCTTGTT, DR3R: 5′- GAACGTGGCCGTTACAGTTTC, DR8R(HHV-6A): 5′-GGATTACGGAGGTGAATGTTGC and DR8R(HHV-6B): 5′-CGCCCGCGACTGCCATAGAG. Control primers in the opposite orientation that did not yield STELA amplicons were: DR1F: 5′-ACCTTGGCCCGAGCAAGAATGC, DR3F: 5′- TCCGTTCCCTCATCGGCATCT and DR8F: 5′-CATAGATCGGGACTGCTTGAA. The DNA in each STELA reaction ranged between 100 and 1000 pg. Agarose gels (0.8%) were used for telomere length analysis with size markers (GeneRuler 1 kb and GeneRuler High Range DNA ladder, Fermentas) run in every gel. Amplicon length analysis was conducted on the phosphor-images (minimum two gels per sample) using the Imagequant software (Typhoon 9400, GE Healthcare). The length of the flanking DNA was subtracted from each amplicon, and the median telomere length and interquartile range of the telomere molecules were determined. For each donor, the data from four telomeres are presented as scatter plots (GraphPad Prism; GraphPad Software Inc., CA, USA). A random sample of blots was selected for independent reanalysis. Within each donor, the median length of the four measured telomeres was ranked to identify the shortest. To determine which telomere was most frequently the shortest across the donors, the shortest telomere in each donor was assigned a value of 1 and the other three telomeres given a value of 0. In donors where the difference between the two shortest telomeres was <100 bp, a value of 0.5 was assigned to each. The shortest telomere frequencies were compared using the non-parametric Kruskal–Wallis analysis (GraphPad Prism), and if *P* < 0.05, the Dunn’s multiple comparison test was used to compare specific telomeres.

### Sequence analysis

Single STELA products were reamplified, gel purified and sequenced by the Sanger dideoxy termination method using DR1R, DR392R 5′-CCAGATGCGAGGATKAGTG; DR421R 5′-GAGKGGTTGAAAGAGGGGTAG or Teltail. Other PCR products were either sequenced directly or, if necessary, reamplified, gel purified and then sequenced.

### Isolation and characterization of chromosomal-HHV-6 junctions

The 10q-CI-HHV-6A junction fragment was isolated by inverse PCR using the primers IPCR-1 and IPCR-2 (25). The isolated DNA fragment was sequenced. The primer 10qF: 5′-ATCCTTCCTCTTTGCAGCCG designed from the sequence was used with the DR8F(A): 5′-GCAGAGACAAAAGTATGCGGAAG primer to verify amplification of the 10q-CI-HHV-6A junction from genomic DNA in this CI-HHV-6A carrier. The other four chromosome-CI-HHV-6-junction fragments were amplified with a primer designed from the 17 p subtelomeric sequences (36). The primer SubT17-539: 5′- CCCAATTTACTGGTAATGGACT anneals to subtelomeric DNA sequences on several chromosomes, and it was used with the DR8F primer to isolate junction fragments from one CI-HHV6A and three CI-HHV-6B carriers. The isolated amplicons were sequenced.

### Sperm DNA analysis

The panel of sperm donors (32) was screened for the presence of HHV-6 as described. There was sufficient DNA to conduct telomere length analysis by STELA on one CI-HHV-6A and three CI-HHV-6B carriers. Telomere length measurements in the sperm DNA samples were made from phosphor-images of STELA blots and scatter plots produced (as above). Frequency histograms were generated by sizing individual molecules and allocating them to 1 kb bins. Distributions were then fitted using a model with either one or two Gaussian distributions (GraphPad Prism). For single Gaussian distributions, outliers were then defined as those telomere lengths of greater or less than three standard deviations from the mean (which includes >99.7% of values from that population). Where the data showed two Gaussian distributions, the distribution of the major population was used to determine the number of outliers. The frequency of short outliers was then determined as a percentage of the total number of telomere molecules analysed and plotted against the mean telomere length. The data were tested for linear correlation using Pearson’s correlation coefficient (GraphPad Prism).

### Analysis of the HHV-6 DR regions

The length of the DR_L_-T2 was determined following PCR using primers UDL6R: 5′-TTTCGCTCACGTGGCAGTCT and DR8F. The presence of PAC2 in these amplicons was determined by PCR with UDL6R and a primer that anneals to PAC2, PAC2F: 5′-TGGGAGGCGCCGTGTTTTTC. The absence of the PAC1 sequence in STELA products (encompassing part of DR_L_-T1 and the telomere) was determined by sequencing reamplified gel-purified products using primers DR1R, DR392R, DR421R or Teltail. The length of the DR_R_-T1 region was determined by PCR amplification between U100F2: 5′-TATCTCCGAACATGATGCTG and DR1R, and the presence of DR_R_-PAC1 was determined by secondary PCR with U100F2 and PAC1RB 5′-CGGGGAGTTTAAAGTAATTTTTG. The presence or absence of PAC1 was confirmed by digestion of STELA products (DR_L_) or U100F2-DR1R amplicons (DR_R_) with *Sma*I (cuts at PAC1) or *Sal*I as a control for digestion.

### Sequencing the CI-HHV-6B genome using ion semiconductor technology

Overlapping amplicons across the unique region of HHV6 and the DRs (without T1 and T2) were generated from CEPH1375.02 CI-HHV-6B carrier, using primers based on the HST sequence (AB021506.1) (37). The amplicons were pooled and sonicated using a Bioruptor (Diagenode Inc.). The subsequent preparation of the sonicated DNA for sequence analysis using an IonTorrent personal genome sequencer (Life Technologies) was essentially conducted according to the manufacturer’s protocol. Briefly, the sheared DNA was end repaired and the adapters were ligated (according to Life Technologies’ protocol). Size selection of ∼170 bp fragment was achieved in a 2.5% NuSieve agarose gel followed by purification using a Zymoclean Gel DNA recovery kit (ZymoResearch, Irvine, CA, USA). The size-selected DNA was then prepared for Ion Torrent sequencing on a 314 chip (100 bp read length) according to Life Technologies’ protocol.

### Detection of HHV-6 RNA transcripts

Total RNA was extracted using Tri-reagent (Sigma-Aldrich). The RNA was dissolved in water (100 µl) and treated with RNase-free DNase I (100 U/µl, 20 min, 37°C). The RNA was purified by phenol–chloroform extraction, precipitated with isopropanol and re-suspended in RNase-free water (100 µl). cDNA was synthesized from 2 µg of total RNA using M-MuLV reverse transcriptase (10 u/µl, New England Biolabs) and random primer hexamers (0.2 µM) in a total reaction volume of 20 µl at 42°C for 1 h, followed by enzyme inactivation at 90°C for 10 min. PCRs were then performed in 10 µl, containing 40 ng of synthesized cDNA and 0.05 U/µl of *Taq* DNA polymerase. Oligonucleotides used were U38F: 5'-TTGTAATTCTGCGCCGTTATC and U38R: 5'-TGCTGGTCCTTCGACATAGA; U73F: 5'-TTACGGAGCGGCAGATAAAC and U73R: 5'-TGATGAGGGCGATTTGAGTA; U90B2F: 5'-CCTGTTTCATGGCAGCCTTC and U90B2R: 5'-ACGACATCGCTTCCAAGAATG for HHV6B or U90A2R: 5'-CCTTCGATGACCTGATTATTCCA for HHV6A; U94-2 F: 5'-AGACAGCCATTCGATGGTTC and U94-1 R: 5'-TGGTACGCTCAAGCGGAGAA. Thermal cycling conditions were denaturation at 96°C for 1 min followed by 35 cycles at 96°C for 15 s, annealing at 58–64°C (depending on amplicons) for 20 s, extension at 70°C for 1 min and a final extension step at 70°C for 10 min. PCR products were analysed by agarose gel (1%) electrophoresis.

### 2D gel analysis

Genomic DNA (25 µg) from LCLs was digested with 100 U of *Eco*RI in a 200-µl reaction volume at 37°C for 2 h, extracted with phenol–chloroform and precipitated with sodium acetate and ethanol. To enrich for circular molecules, the *Eco*RI-digested genomic DNA was then digested with 100 U of ‘Plasmid-safe’ ATP-dependent DNase (Epicentre), which only digests double-stranded linear DNA, in a total volume of 500 µl at 37°C for 20 h. The DNA was then concentrated by isopropanol precipitation before 2D gel electrophoresis. Briefly, the DNA was separated in 0.4% agarose, 0.5× Tris-borate-ethylenediaminetetraacetic acid buffer, pH 8.3, at 1 V/cm for 18 h at 4°C in the first dimension and in 1% HGT agarose in 0.5 × Tris-borate-ethylenediaminetetraacetic acid containing 0.5 µg/ml ethidium bromide at 5 V/cm for 5.5 h in the second dimension. A Southern blot was prepared and hybridized to a ^32^P-labelled HHV6B DR probe, then after stripping to a telomere probe.

### Detection of CI-HHV-6B truncations at DR_L_-T2

Genomic DNA (0.5 µg) was digested with *Exo*I (New England Biolabs) to remove 3′ single-strand overhangs from telomeres. Following *Exo*I digestion, the integrity of the double-stranded genomic DNA was verified by PCR amplification of ∼6 kb fragment using primers U100FB 5'-CATCTGATCGTCATTTGGCG and DR1R. STELA was conducted as above using flanking primers DR1R or the UDL6R on genomic DNA with or without *Exo*I digestion.

### Detection of extra-chromosomal circular HHV-6 DNA

Genomic DNA (90 ng/reaction) was amplified with primers UDL6R and U100F at 0.2 µM with Taq (0.04U/µl) and *Pwo* (0.025U/µl) DNA polymerases in 10 µl reaction prepared in the same PCR buffer used for STELA. Control PCRs were also performed with primers UDL6R and DR3F or U100F2 and DR3R. The PCR products were resolved in 0.8% agarose gels, and Southern blots were prepared and hybridized to a DR3 probe generated using DR3F and DR3R primers. The amplicons generated from extra-chromosomal circular DNA in five separate PCRs from CEPH1375.11 CI-HHV-6B were reamplified and the composition of the DR region was verified by secondary PCRs and sequencing.

## RESULTS

### Identification of CI-HHV-6 carriers and verification of viral integration using STELA

**Figure 1.**
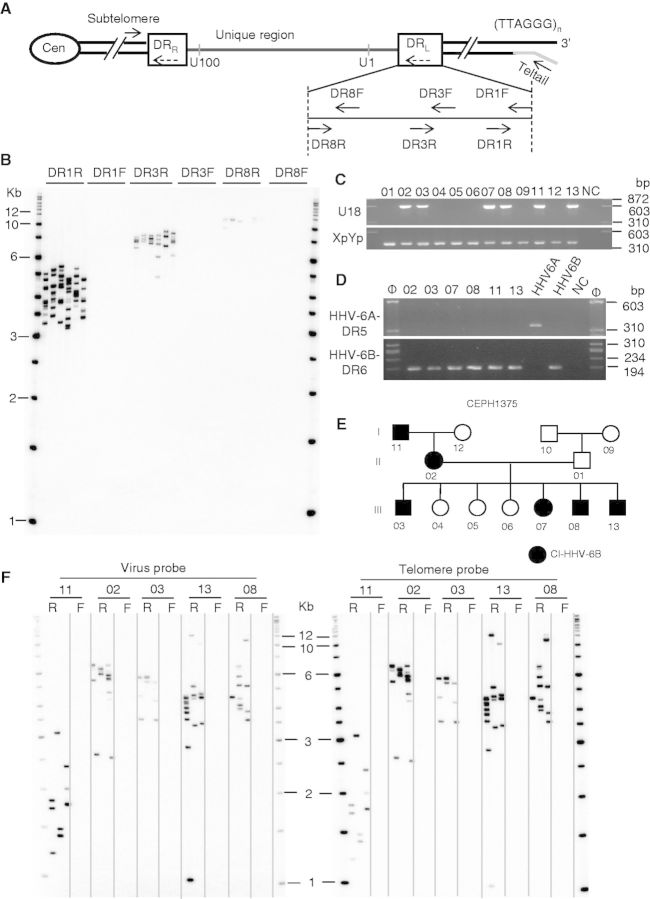
Characterization of CI-HHV-6 carriers and proof of telomeric integration using STELA. (**A**) The HHV-6A or 6B genome inserts in the reverse orientation such that DR_R_ and the U100 gene are closest to the subtelomeric sequences. The DR_L_ is expanded to show the reverse order of DR8, 3 and 1 ORFs. Plain arrows represent primers. The telorette 2 oligonucleotide (grey line) and Teltail primer used in STELA are shown. Orientation of DRs is shown by dashed arrows. Cen, centromere. (**B**) Amplification of the CI-HHV-6-associated telomere using STELA in a CI-HHV-6B carrier [1-9p34.3 (24)]. Amplicons are generated with the DR1, 3 and 8 reverse (R) primers but not the forward (F) primers, confirming the orientation of the integrated virus. The increasing size of the STELA products generated by DR1R, 3R and 8R reflects the distance from the 3′ single-strand overhang. The blot was hybridized to a telomere probe. **(C–E)** Identification of the CEPH1375 family that segregates CI-HHV-6B through three generations. (**C**) Family members were screened for the presence the HHV-6 U18 gene. An amplicon from the XpYp telomere-adjacent sequence was used as a PCR control. (**D**) CEPH1375 family members are positive for CI-HHV-6B and showed the presence of the HHV-6B DR7 and absence of the HHV-6A DR5. Φ, size marker; NC, negative control. **(E**) CEPH1375 family showing transmission of CI-HHV-6B through three generations. (**F**) Detection of STELA amplicons using the DR1R (R), but not the DR1F (F), primer confirmed the orientation and integration of HHV-6B in family members. All the STELA amplicons contain viral sequences (left) and (TTAGGG)_n_ repeats (right). The maternal grandfather (CEPH1375.11) shows particularly short telomeres on the end of the CI-HHV-6B.

To determine whether HHV-6 integration is a natural biological form of latency and what effect it has on the associated telomere, we investigated six LCLs from unrelated CI-HHV-6 carriers (24) with integration sites as follows: one CI-HHV-6 A at 10q, two CI-HHV-6B at 9q, one CI-HHV-6B at 11 p and two CI-HHV-6B at 17 p (24). We used STELA (33) to confirm integration and to orientate the viral genome within the telomere (Figure 1A and B).

To identify more CI-HHV-6 carriers, we screened 3859 DNA samples from unrelated donors (see Materials and Methods) using primers that amplify conserved segments from the viral U11 and U18 genes. Integration was verified using STELA. As a result, 58 additional CI-HHV-6 positive samples were identified among the various populations (4 CI-HHV-6 A and 54 CI-HHV-6B). Where available we obtained LCLs or additional high molecular weight genomic DNA from the newly identified CI-HHV-6 carriers for further analysis. We also identified a family (CEPH 1375) that has transmitted CI-HHV-6B over three generations (Figure 1C–F).

### Characterization of the DRs in CI-HHV6 carriers

**Figure 2.**
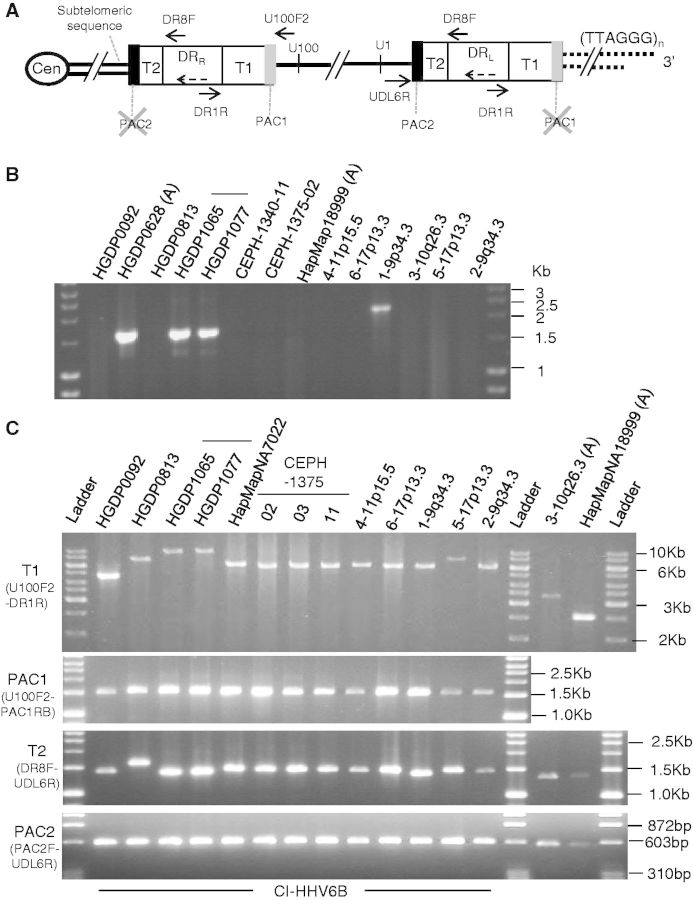
Characterization of the viral DRs in CI-HHV-6 carriers. (**A**) Diagram summarizing the composition of DR_R_ and DR_L_ in CI-HHV-6A and B carriers. The expected location of the cleavage and packaging sequences, PAC1 and 2, is shown. The terminal PAC1 and PAC2, which are absent in CI-HHV-6 carriers, are identified (grey cross). The orientation of the DRs is shown by dashed arrows; plain arrows represent primers. (**B**) Isolation of internal subtelomere-CI-HHV-6 junctions in one CI-HHV-6A carrier (HGDP00628) and three CI-HHV-6B carriers (HGDP01065, HGDP01077, both Sardinians and 1-9p34.3) by PCR amplification between a subtelomeric primer (SubT17-539) and a primer in DR8 (DR8F). The subtelomere-CI-HHV-6 junctions in the other nine CI-HHV-6 samples analysed did not amplify with the SubT17-539 primer. (**C**) Characterization of viral T1 and T2 telomere-like repeat regions and their associated PAC1 and PAC2 sequences. PCR amplification across the DR_R_-T1 region of degenerate telomere-like repeats using a primer anchored in the unique region (U100F2) with the DR1R primer in 13 CI-HHV-6B and 2 CI-HHV-6A carriers shows that T1 is greatly expanded (particularly in CI-HHV-6B) and varies in length between carriers (top). The DR_R_-PAC1 sequence is retained in all CI-HHV-6B carriers, as shown by secondary PCR using the PAC1RB primer (specific to HHV-6B PAC1) with the U100F2 primer (second panel). The DR_L_-T2 region [pure (TTAGGG)_n_] shows the expected modest length variation between carriers, as demonstrated by PCR with a primer anchored in the unique region, UDL6R, and DR8F (third panel). In addition, the DR_L_-PAC2 sequence is retained in all carriers, as shown by secondary PCR using the PAC2F primer with UDL6R (bottom).

Sequence analysis of single STELA products from seven individuals showed that the terminal PAC1 sequence (at the distal end of DR_L_) was absent in CI-HHV-6 A (*n* = 2; 3-10q26.3 and 1501) and CI-HHV-6B (*n* = 5; 1-9q34.3, 2-9q34.3, 5-17p13.3, 6-17p13.3 and HGDP0092) carriers. We isolated the 10q chromosome-HHV-6 junction from one CI-HHV-6 A carrier [3-10q26.3 (24)] using inverse PCR (25). The internal chromosome-HHV-6 junctions from four other carriers were isolated by amplification between a subterminal sequence (found at several chromosome ends, Sub17p-539) and the DR8F primer in DR_R_ (Figure 2 and Supplementary Figure S1). The isolated chromosome-HHV-6 junctions all lacked the terminal PAC2 sequence and showed a similar organization with a short subterminal sequence, TTAGGG and sequence-variant repeats commonly found in human telomeres (3) followed by more (TTAGGG)_n_ repeats of viral or human origin and the DR8 sequence. In summary, the two CI-HHV-6 A junction fragments (from 3-10q26.3 and HGDP0628) and two of the three CI-HHV-6B junction sequences (from 1-9q34.3 and HGDP1065/HGDP1077) are different from one another (GenBank Accession Numbers: KF366418, KF366419, KF366420). The two Sardinian CI-HHV-6B carriers (HGDP1065 and HGDP1077) carried identical junctions suggesting common ancestry.

Further characterization of the left and right DR regions in 15 CI-HHV-6 carriers using a variety of primers (some anchored in the unique region to facilitate specific amplification of DR_R_ or DR_L_) confirmed that the sequence organization is the same between the DRs. The length of the T2 region (perfect (TTAGGG)_n_) in DR_L_ is variable between carriers (20). Unexpectedly, the T1 region (degenerate telomere-like repeats) in DR_R_ is highly expanded compared with the published strains (HHV-6 A U1102; HHV-6B HST and Z29) ranging between 0.7 and 1.5 kb in CI-HHV-6 A carriers and more strikingly 3–9 kb in CI-HHV-6B carriers (Figure 2C). We also show that all CI-HHV-6 carriers retain a copy of the PAC2 sequence immediately adjacent to DR_L_-T2 and an internal copy of PAC1 immediately distal to DR_R_-T1 (Figure 2C and Supplementary Figure S2).

### The CI-HHV6 telomere was often the shortest measured in carriers

**Figure 3.**
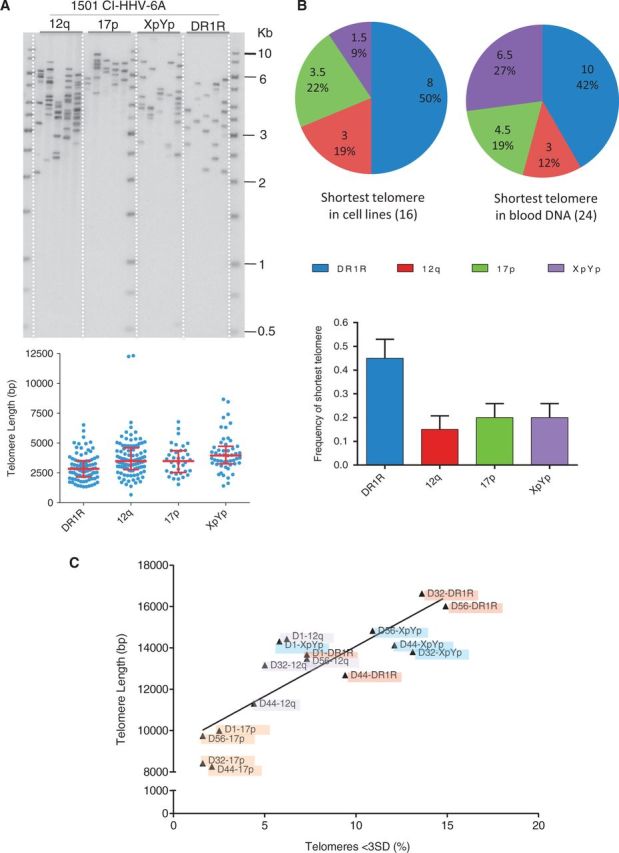
Telomere length analysis in CI-HHV-6 carriers. (**A**) STELA was used to measure telomere length at 12q, 17 p, XpYp and on the end of the virus (DR1R) in CI-HHV-6 carriers. It shows a representative STELA-Southern blot on blood DNA from one CI-HHV-6 A carrier (1501) and below the scatter plots of the data obtained from several blots. The median and inter-quartile ranges are shown as red lines. (**B**) The median values for each of the four telomeres measured were ranked by length in DNAs from 16 LCLs and 24 blood DNAs from CI-HHV-6 carriers. The proportion of the samples in which each telomere is the shortest is shown in pie charts for the LCLs and blood DNA samples. The histogram of the combined data shows that the shortest telomere frequency (+SEM) is different between the chromosome ends (Kruskal–Wallis test *P* < 0.0117). (**C**) Relationship between telomere length and the frequency of short telomere molecules in the male germline. The short (outlier) telomere molecules, shorter than 3 standard deviations from the mean (<3SD), were counted as a proportion of the total molecules analysed for each telomere in four sperm donors and plotted against mean telomere length (Table1).

To investigate the length of the telomere on the end of the virus, we used STELA with the DR1R primer to amplify telomere molecules from small aliquots of LCL-DNA from CI-HHV-6 carriers (Figure 3A). The median length of the virus-associated telomere was compared with the length of XpYp, 12q and 17 p telomeres. In 50% (8/16) of the LCLs, the CI-HHV-6-associated telomere was the shortest measured (Figure 3B, Supplementary Figure S3A) (9,38), even compared with the 17 p telomere that has been reported as often being the shortest (36,39). Corresponding analysis of blood DNA samples from 24 carriers (21 CI-HHV-6B from the Orkney Complex Disease Study study, and 3 CI-HHV-6 A siblings in a British family) again showed that the virus-associated telomere was most often the shortest [42% (10/24); Figure 3B and Supplementary Figure S3B], indicating that short virus-associated telomeres do not arise from the establishment or propagation of LCLs. The combined data show that the shortest-telomere frequency differs significantly between the chromosome-ends in somatic cells (Kruskal–Wallis test *P* < 0.0117) and is highest at the CI-HHV-6-associated telomere (shortest in 45% of the carriers).

### CI-HHV-6-associated telomeres in the germline

To investigate virus-associated telomere lengths in the germline, we screened sperm DNA samples from 92 men from UK of North European descent (32) and identified five (5.4%) CI-HHV-6 carriers (4 HHV-6B and 1 HHV-6 A), a higher frequency than seen in the British Isles panel but not statistically significant. Telomere length analysis in four men showed that all were considerably longer in sperm DNA, as expected (40). Interestingly, the 17 p telomere, not the CI-HHV-6-associated telomere, was the shortest we measured in the germline of all four sperm donors. Moreover, the CI-HHV-6-associated telomere was the longest measure in two of the sperm donors (Table 1 and Supplementary Figure S4).

**Table 1.** Telomere length analysis in sperm DNA from CI-HHV-6 carriers

Donor and telomere	Mean length ± SD (bp)	Total number of bands	Number of outliers	Percentage outliers
D1-DR1R	13 676 ± 1950	385	28	7.3
D1-12q	14 428 ± 4111	357	22	6.2
D1-17p	9996 ± 1825	277	7	2.5
D1-XpYp	14 311 ± 2931	259	15	5.8
D32-DR1R	16 625 ± 2627	191	26	13.6
D32-12q	13 160 ± 2970	261	13	5.0
D32-17p	8424 ± 1719	256	4	1.6
D32-XpYp	13 803 ± 1418	153	20	13.1
D44-DR1R	12 680 ± 2172	255	24	9.4
D44-12q	11 304 ± 3085	482	21	4.4
D44-17p	8259 ± 1535	187	4	2.1
D44-XpYp	14 122 ± 1210	141	17	12.1
D56-DR1R	16 018 ± 1600	141	21	14.9
D56-12q	13 470 ± 3569	358	26	7.3
D56-17p	9742 ± 2427	243	4	1.6
D56-XpYp	14 825 ± 1201	156	17	10.9

Examination of the telomere length distributions in the sperm samples showed that a proportion of outlier molecules was considerably shorter than the mean length, as reported previously (40). The shortened telomere molecules likely arise through intra-telomere trimming via the t-loop excision mechanism (7,41-43). We showed that the frequency of short outlier molecules correlated with mean telomere length in the germline. Consequently, the longest CI-HHV-6-associated telomeres have the highest frequency of outliers (Table 1 and Figure 3C), indicating a high frequency of t-loop excisions within the telomere.

### The HHV-6-associated 12q, 17 p and XpYp telomeres shorten at similar rates in LCLs

The difference in the ranked length of the CI-HHV-6-associated telomere between somatic cells and the germline suggests that the presence of the viral genome affects length regulation. Analysis of telomere shortening rates in two LCLs (GM18999 CI-HHV-6 A and CEPH1375.02 CI-HHV-6B) showed similar attrition rates (average 79 bp/cell division) at the Xp/Yp, 12q, 17 p and CI-HHV-6-associated telomere in both cell lines (Supplementary Figure S5). This suggests that the disparity between the length of the CI-HHV-6-associated telomere in somatic cells and the germline is not explained by a higher rate of telomere erosion at the virus-associated telomere. However, we detected low-level telomerase expression in the CEPH1375.02 CI-HHV-6B at all time points and in some time points for the GM18999 CI-HHV-6A cell line. Clearly, the level of telomerase is insufficient to maintain telomere length in these cell lines but we cannot exclude the possibility that it may target one telomere more than another.

### Somatic truncation of the integrated HHV-6 genome and viral excision

**Figure 4.**
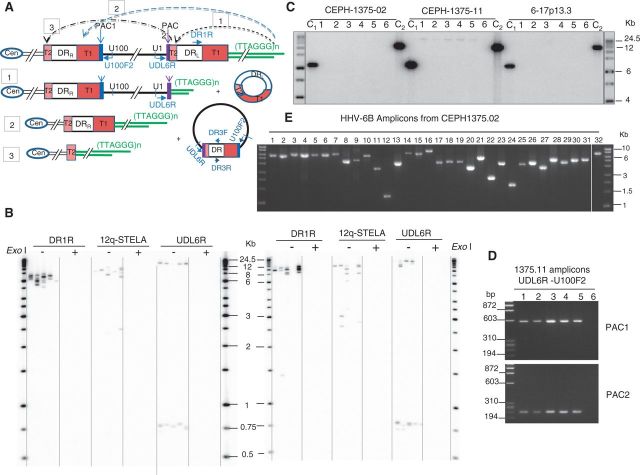
The integrated viral genome can be truncated, leading to the formation of short telomeres at new locations and to the release of circular DNA molecules that contain viral sequences. (**A**) Truncation events [1–3] that could arise via t-loop formation between the telomeric 3' single-strand overhang and viral T1 or T2 regions and the circular molecules that could be released. Truncation at DR_L_-T2 [1] could arise through t-loop formation between the telomere and DR_L_-T2, and small circular molecules comprising one DR region could be released. T-loop formation between the telomere and the internal DR_R_-T1 [2] or between a chromosome that had already undergone a truncation at DR_L_-T2 and the internal DR_R_-T2 [3] could be cleaved to release a large circular molecule composed of the entire viral genome and a single DR region reconstituted with both PAC1 and PAC2. The reciprocal product would be a chromosome that retains a single DR (without PAC1 or PAC2) plus a telomere [2] or the complete removal of the viral sequence from the chromosome [3]. (**B**) Detection of CI-HHV-6 molecules truncated at DR_L_-T2 in two CI-HHV-6B carriers [CEPH1375.02, left and 4-11p15.5 (24), right]. Telomeres were detected at the end of the full-length virus using primer DR1R and at 12q (12q-STELA), as expected. The amplicons generated from a primer located near the U1 gene (UDL6R) fell into two clusters. Amplicons ∼750 bp in length were derived from truncated CI-HHV-6 molecules with short telomeres at DR_L_-T2. The larger products generated with the UDL6R primer are equivalent to the products from the DR1R primer. STELA products were only seen in the DNA without *Exo*I digestion, showing they are dependent on the presence of a telomeric 3' single-strand overhang. (**C**) Detection of molecules that encompass the HHV-6B unique region and a single DR with both PAC1 and PAC2. Amplification of multiple aliquots of genomic DNA (90 ng/reaction) from CEPH1375.11 (grandfather) using the primers UDL6R and U100F2 (shown in A) that are only directed towards one another on circular DNA molecules, generated large amplicons in five of the six PCRs shown but not from other CI-HHV-6B carriers in the same family (CEPH 1375.02, mother shown) or in an unrelated CI-HHV-6B carrier (6-17p13.3). Control amplicons, C_1_ (UDL6R + DR3F) and C_2_ (U100F2 + DR3R), were generated in all the samples. The amplicons were detected by hybridization to a probe for DR3. (**D**) The amplicons generated from circular HHV-6 DNA molecules in CEPH1375.11 contain a single DR with both PAC1 and PAC2. Specific amplifications show the presence of PAC1 and PAC2 in each amplicon generated in (C). (**E**) The complete HHV-6B genome is present in the CEPH1375 family. Overlapping amplicons (1–31) covering the unique portion of the HHV-6B genome and the DRs (amplicon 32, without T1 and T2) were generated from CEPH1375.02. Each amplicon is the expected size showing there are no gross rearrangements of the integrated viral genome.

The detection of short telomere molecules in the germline (Supplementary Figure S4 and Figure 3C) (40) prompted us to look for extra-chromosomal circular molecules. We detected extra-chromosomal circles containing (TTAGGG)_n_ repeats in the CEPH1375.02 LCL, which arise from low-frequency intra-telomere t-loop excision at all telomeres (41–43). We also detected extra-chromosomal circular molecules containing HHV-6 DR sequences (Supplementary Figure S6). These may arise from excision of a quasi t-loop formed by invasion of the telomeric 3′ single-strand overhang into the DR_L_-T2 (Figure 4A). The reciprocal product of excisions should be a truncated CI-HHV-6 with a novel telomere at DR_L_-T2. Therefore, we conducted STELA using a flanking primer at the U1 gene and detected very short telomere molecules (140–200 bp) in five CI-HHV6B cell lines (two shown in Figure 4B). Amplification of the short telomere molecules is dependent on the presence of a telomeric 3′ single-strand overhang, as *Exo*I digestion before STELA abolished the products. Sequence analysis showed these amplicons contain DR_L_-PAC2.

We hypothesized that the entire viral genome could be released from the telomere through telomeric 3′ single-strand overhang (eroded into the DR_L_-T1 region) invasion into the internal DR_R_-T1 region or between a viral genome already truncated at DR_L_-T2 into the internal DR_R_-T2 (Figure 4A). Resolution of the quasi t-loop, which would include the whole viral genome, could release the HHV-6 genome as a large circular molecule comprising the unique region and a single reconstituted DR with both PAC1 and PAC2. Telomere length analysis in the CEPH1375 family had shown that the telomere on the distal end of CI-HHV-6B was particularly short in the maternal grandfather, CEPH1375.11 (Figure 1F). In addition, truncated CI-HHV-6B molecules with a short telomere at DR_L_-T2 were readily detected in CEPH1375.11 suggesting frequent abnormal t-loop formation and excision events in this carrier. Amplification of CEPH1375.11 genomic DNA with the viral primers, UDL6R (in the U1 gene) and U100F2 (Figure 4A), that point away from one another on the integrated virus generated large products (∼16 kb) that were detected by Southern blot hybridization to the viral DR3 or (TTAGGG)_n_ probe (Figure 4C). Characterization of these products showed they include U1, PAC2, T2, DR8 to DR1, T1 (∼5 kb), PAC1 and U100 sequences (Figure 4D), indicating they contain a reconstituted DR region with both PAC1 and PAC2. The ∼16-kb amplicons generated from CEPH1375.11 are not artifacts arising from jumping-PCR, as no such amplicons were generated from genomic DNA of other CI-HHV-6B carriers in the CEPH1375 family (CEPH1375.02 shown) or from an unrelated CI-HHV-6B carrier (6-17p13.3).

### Integrated HHV-6B genome is intact in the CEPH1375 family

If telomere integration is an effective form of latency (44), then the viral genome should remain intact despite localization adjacent to subtelomeric regions that have a relatively high turnover rate (45). To investigate this in the CEPH1375 family, we generated overlapping amplicons from the HHV-6B unique region and DRs (without T1 and T2) from CEPH1375.02. All amplicons were the expected sizes (Figure 4E). Similar analysis in a further 11 unrelated CI-HHV-6B carriers also showed that the integrated viral genomes lacked any gross rearrangements. The HHV-6B genome in CEPH1375.02 was sequenced using ion semiconductor technology (37), with small gaps closed using Sanger sequencing. This confirmed that the integrated HHV-6B genome in CEPH1375.02 is intact and differs from the HHV-6B HST strain by 282 base substitutions and a few single base indels. *In silico* analysis of the base substitutions and indels indicates that none are predicted to cause disruptive frameshifts in the ORFs (Supplementary Figure S6).

### Spliced U90 transcripts detected in CI-HHV-6 carriers

**Figure 5.**
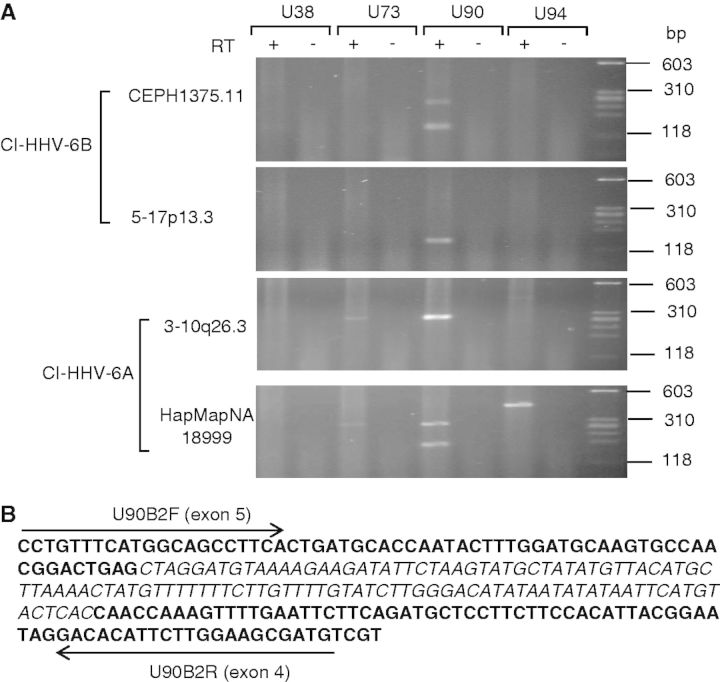
Detection of viral transcripts from CI-HHV-6. (**A**) RT-PCR was used to detect transcripts from U38, U73, U90 and U94 in LCLs from two CI-HHV-6A and eight CI-HHV6B carriers. Two different U90-RT-PCR products were visible in RNA samples from seven LCLs (e.g. CEPH1375.11 and HapMapNA18999). These products were of the expected sizes for unspliced and spliced transcripts. (**B**) Sequence analysis of the shorter U90 RT-PCR product showed that the intron (110 bp in italics) between exons 4 and 5 (44) was absent confirming that the U90 RNA had undergone correct splicing. Black arrows show the positions of the U90B2F and U90B2R primers.

Expression analysis of U38, U73, U90 and U94 in 10 different CI-HHV-6 LCLs consistently revealed transcripts from the U90 gene in all 10 LCLs, whereas detection of transcripts from other genes varied between the LCLs and between RNA samples. Seven LCLs showed two different length U90-reverse transcriptase PCR (RT-PCR) products, two LCLs showed only the shorter product and one LCL showed only the longer product. Sequence analysis showed that the shorter product had undergone a correct splicing to remove an intron (Figure 5). The HHV-6 U90 gene is an immediate early transactivator that produces different transcripts during productive infection and latency owing to alternative splicing and the use of alternative start sites (46). Detection of the spliced U90 transcripts is further evidence that CI-HHV-6 is a form of latency.

## DISCUSSION

Among the five internal CI-HHV-6 junction fragments we isolated (2 CI-HHV-6A and 3 CI-HHV-6B carriers), four were different, thus representing independent integration events, and all shared a similar sequence organization (25,47). The absence of DR_R_-PAC2 and the presence of telomere sequence-variant repeats (3) in each junction support the proposition that HHV-6 integrates by HR between DR_R_-T2 and the proximal end of a telomere. The absence of PAC1 at the terminal end of DR_L_ could also be a consequence of HR at the time of integration. However, the curious expansion of the T1 region, identified through analysis of the internal DR_R_-T1, and the variable truncation of the terminal DR_L_-T1 in carriers suggest that the degenerate repeat region underwent erosion. Therefore, the expanded T1 region may serve a role during integration by acting as a buffer for replication-driven erosion that results in loss of PAC1 and part of the DR_L_-T1 region until (TTAGGG)_n_ repeats are added by telomerase in the germline.

The CI-HHV-6-associated telomere was the shortest measured in somatic cells from 45% of the 40 carriers investigated but not the shortest in the four CI-HHV-6 sperm donors showing that the virus-associated telomere is readily lengthened by telomerase in the germline. The disparity between CI-HHV-6-associated telomere length in somatic cells and the germline suggests that the integrated virus perturbs an aspect of telomere function. We showed that the rate of telomere erosion through replication-driven processes was not higher at the CI-HHV-6-associated telomere in LCLs from two CI-HHV-6 carriers. However, as these cell lines showed a low level of telomerase activity, further analysis will be required to determine whether or not telomerase is targeted to the CI-HHV-6 telomere more frequently.

We surmised that the presence of the HHV-6 genome might interfere with telomere capping by perturbing of t-loop formation and excision giving rise to telomere rapid deletion events that were first described in yeast (48,49). We showed that LCLs from CI-HHV-6B carriers are mixed populations of cells carrying the full-length CI-HHV-6B and a subset with CI-HHV-6B chromosomes truncated at DR_L_-T2 by the presence of a novel very short telomere with a single-strand overhang (Figure 4). The truncations at DR_L_-T2 could arise through a double-strand break at DR_L_-T2, followed by end processing to generate a 3′ single-strand overhang. However, as we also detected extra-chromosomal circular molecules containing HHV-6 DR sequences, we favour a model in which the truncated chromosomes arise through processing of a t-loop (41–43) formed by strand invasion of the telomeric 3′ single-strand overhang within DR_L_-T2. Excision of the quasi t-loop would result in a sudden truncation and formation of a telomere at DR_L_-T2. The presence of the novel short telomere may contribute to cell cycle arrest and the onset of senescence.

Sequence analysis of the CI-HHV-6B genome in the CEPH1375.02 showed that the viral genome is intact in this family; moreover, we detected molecules that contain a reconstituted DR region with both PAC1 and PAC2 in the grandfather CEPH1375.11. The reconstituted DR region could only arise from a recombination event between the terminal DR_L_ (that contains a full-length T2 and PAC2) and the internal DR_R_ (that contains a full-length T1 and PAC1) resulting in the release of a circular molecule containing the viral genome with a single DR (Figure 4). Therefore, consistent with our model invoking t-loop formation including the viral genome, we propose that occasional single-strand invasion of the telomeric 3′ overhang into the internal HHV-6 DR_R_-T1 facilitates release of the entire viral genome from the chromosome with a single reconstituted DR (Figure 4A). These large extra-chromosomal circular molecules arise from an intact viral genome (as shown by the next-generation sequence analysis), and so they have the potential to undergo rolling circle replication that could regenerate full-length HHV-6B genomes with two identical terminal DRs. This and the evidence that spliced transcripts from the U90 gene, which is involved in latency, were detected in the cell lines investigated support the hypothesis that CI-HHV-6 is an alternative form of viral latency.

The reciprocal product of a large excision event would be retention of a DR, lacking both PAC sequences, within the telomere. Interestingly, we have identified two unrelated individuals (2/3859; population frequency 0.05%) that lack the unique portion of the HHV-6B genome but carry a single HHV-6B-DR (without PAC1 and 2) integrated into a telomere. These individuals may have inherited chromosomes that had already undergone a viral excision event, although partial insertion during integration cannot be excluded. The released viral genome with a single reconstituted DR in CEPH1375.11 could also arise by strand invasion of a short telomere on a CI-HHV-6 molecule already truncated at DR_L_-T2. Excision would release a circular HHV-6 genome with a reconstituted DR and remove the viral genome from the telomere (Figure 4A [3]).

In summary, we have shown that the CI-HHV-6-associated telomere is often one of the shortest in somatic cells and prone to sudden deletions that create a critically short telomere at a new location in the viral genome. The presence of a short telomere associated with the CI-HHV-6 will increase the chance that the cell will become senescent, thus affecting tissue homeostasis (9,10). Moreover, cells carrying critically short telomeres associated with CI-HHV-6 may be prone to telomere fusion events that can drive instability. We also show that integrated copies of HHV-6B can be excised from the chromosome and we propose that this is achieved through use of the t-loop excision mechanism. This may facilitate spreading to another telomere within the cell or represent the first step towards viral reactivation.

## ACCESSION NUMBERS

GenBank: KF366418, KF366419, KF366420.

## SUPPLEMENTARY DATA

Supplementary Data are available at NAR Online.

## FUNDING

The Medical Research Council [G0901657 to N.J.R.]; the Wellcome Trust Institutional Strategtic Support Fund [WT097828MF] and by Conacyt, Mexico (to A.H.-B.). Funding for open access: University of Leicester RCUK.

*Conflict of interest statement*. None declared.

## Supplementary Material

Supplementary Data
