# Supplementary material for: Human telomeres that carry an integrated copy of human herpesvirus 6 are often short and unstable, facilitating release of the viral genome from the chromosome
Source: Nucleic Acids Res. 2013 Sep 19;42(1):315–27. doi: 10.1093/nar/gkt840 (PMC3874159; doi:10.1093/nar/gkt840)
Supplement: Supplementary Data [file supp_42_1_315__index.html]

Human telomeres that carry an integrated copy of human herpesvirus 6 are often short and unstable, facilitating release of the viral genome from the chromosome — Human telomeres that carry an integrated copy of human herpesvirus 6 are often short and unstable, facilitating release of the viral genome from the chromosome — Supplementary Data 

# Human telomeres that carry an integrated copy of human herpesvirus 6 are often short and unstable, facilitating release of the viral genome from the chromosome

## Supplementary Data

files

**Files in this Data Supplement:**

- Supplementary Data - pdf file
