# Supplementary material for: Human telomeres that carry an integrated copy of human herpesvirus 6 are often short and unstable, facilitating release of the viral genome from the chromosome
Source: Nucleic Acids Res. 2013 Sep 19;42(1):315–27. doi: 10.1093/nar/gkt840 (PMC3874159; doi:10.1093/nar/gkt840)
Supplement: Supplementary Data [file supp_gkt840_nar-02061-h-2013-File007.pdf]

A. Internal chromosome-HHV-6A junction of 3-10q26.3

GATCCTTCCTCTTTGCAGCCGAGAATAATGAGGGTTGGGGTTAGGGTTAGGGTTTAGGGTTTAGGGTTTAG
GGTTAGGGTTAGGGTTAAGGGTTAGGGTTAAGGGTTAGGGTTAGGGTTAGGGTTAGGGTTAGGGTTAGGGT
TAAGGGTTAGGGTTAAGGGTTAGGGTTAGGGTTAGGGTTAGGGTTAGGGTTAGGGTTAGGGTTTAGGGTTT
AGGGTTAGGGTTAGGGTTAGGGTTAGGCTTAGGCTTAGGGTTAGGGTTAGGGTTAGGGTTAGGGTTAGGGT
AGGGTTAGGGTTAGGGTTAGGGTTAGGGTTAGGGTTAGGGTTAGGGTTGTTAGGGTTAGGGTTAGGGTTAG
GGTTAGGGTTGGGGTTGGGGTTGGGGTTGGGGTTGGGGTTGGGGTTGGGGTAGGGGTAGGGGTAGGGGTAG
GGTTGGGGTTGGGGTTGGGGTTGGGGTTGGGGTTGGGGTTGGGGTTAGGGTTAGGGTTAGGGTTGGGTTAG
GGTTAGGGTTAGGGTTAGGGTTAGGGTTAGGGTTAGGGTTAGGGGTTAGGGGTTAGGGGTTAGGGTTAGGG
TTAGGGTTAGGGTTAGGGTTAGGGTTAGGGTTAAGGGTTAGGGTTAAGGGTTAGGGTTAGGGTTAGGGTTA
GGGTTAGGGTTAGGGTTAGGGTTAGGG*TTAGCCGGCGGTTTCCTTTGGCACCGTGCCAGGCAATCGTCGGT*
CCCAGACGGTGGCACGTCGCGCGATGTCGTTCTGAATTGTCTTCAAGTCTCACCCCGGTATGCCCCATC
TACGTACCCACCCGCGACTACCACATGGGTGGGTTGTGCGTGTTTGTGTGTCTGCGTGTGTACGCGTCAGT
GGTAGAAACGCGGAGACAACGGATTACGGAGGTGACTGTTGCGTTGGGTGGGACGGGGAGGGGGTGAAGAG
GTGTTTCTCGTATAGGTGTTTTTGGAACGTGGTTTTTCTTTCTTATACTTGCCATGCTAGCGGTCGCCCTC
TGTCTTCGCTCCGCGTGACCGTGTCGTGTGTACTTCCGCATACTTTTGTTTTTGCGCCTCGATACACCTCG
TTGATTGGATGATTGCCACATGTGTATTCGATGTCACAGTCTGCTATTTTTTTTTCACTGTCTATTTTTAA
ACCTGTTGTCGTCTTCCCGCCAAGGCTGTGCGTCTCCGCCTTTTATTATTTTTTTTACGCTGTTTTTTTT
CTACGTTCGCCTCCAACTCCGGACGCACCTCCTTTGTATGTCGACTCCTCCCACTCCCCGGGGTGTCTATA
TAAGAGGCCAACGCGACGCGCTTTCCAGCAGTCCCGATC

B. Internal chromosome-HHV-6B junction of 1065 Sardinian

GAGCATAGAGGTATTGTTTTCTGTCTGGACTCCCAATTCAATTCAGTTGATCTTTCTGTTTATTCCTGTGC
AAGGATCCCACTGTTTTTATTACTGTTCCTTTGTAATAAAATTTGAAATTGGGATGTGATCAGGATCAGCT
TATCCACTTCTGTCCCAAGGCCTTTGGGATTTTTGTAGGAATAACATCGAATCCACGGATTGCTTTGTGTA
CTTTGGGAAACTTAACAATGTGGTCTACAAATCCACAAATAAGATACATTTTTACATTTACTGGAAGTTTA
ATTTCCTTAAGTAATGTCTTATAATTTCCCTCATCTAAGTCTTGTCGTTTCATTCCATTTATTCCTAAGTA
TAATATTGCTATTGGTATTGTTTAAGGTAGAATTTTCATAATTTGGTTTAGAGATTATTCATTCCTAGCAT
ATACATATAAAATGGAATGTTTGGCTAGGCACCCGGGCTCATACCTGTAACCCAAGCAGGTTGAGAGGCTG
AGGAAGGGTTAGGGTTAGGGTTGGGGTTAGGGTTAGGGTTAGGGTTAGGGTTAGGGTTAGGGTTGGGGTTG
GGGTTGGGGTTGGGGTTGGGGTTGGGGTTGGGGTTGGGGTTGGGGTTGGGGTTGGGGTTGGGG*TGAGGGTG*
*AGGGTGAGGGTGAGGGTGAGGGTGAGGGTGAGGGTGAGGGTGAGGGTGAGGGTGAGGG*TTAGGGTTAGGGT
TAGGGTTAGGG*TGAGGGTGAGGGTGAGGGTGAGGGTGAGGGTGAGGGTGAGGG*GTAGGGTTAGGGTTAGGG
TTAGGGTTAGGGTTAGGGTTAGGG*TGAGGGTGAGGGTGAGGGTGAGGGTGAGGGTGAGGGTGAGGGTGAGG*
*G*TTAAGGGTTAGGGTTAGGGTTAGGGTTAGGGTTAGGGTTAGGGTTAGGGTTAGGGTTAGGGGTTAGGGTT
AGGGTTAGGGTTAGGGTTAGGGTTAGGGTTAGGGTTAGGGTTAGGGTTAGGG*TTATAGATTGCGCCAGAGG*
CTTCCCTTGGCGTCGTGCCAGAATAGCAAGGGTCCGAGACGACGGCACGTCGCGCGCTATGCCGTTCCTCA
TTGACTTTACATCTGCCCCCCGTATGCTCCCCGCCTGCCCGCCCGCGACTGCCATAGAGGTGGTGTGTGCG
TGTCTGCGTGTCTGCGTGTGCACGCGCCTGTGGGAGAAACGCGGAGAGAAGGGATTACGGAGGGGGGGTAT
TGTGGTAGATGGGGTAGGGAGTGGGGTGAAGGATGTTTCCTTTGTTAGTATTTTGCAGCGCTGCTTATTT
TTTTTTCCTAGTTGCCATGCTAGGAGTCGGCCTTTGTCGTCGGTCCGCGTGACTGTCTGTCGCATGCACTT
CCGTATACTTTTTTTTGCGCCCCGGCACGTCCCGTTGATTGGATTGCCGCCGCACGNGTATTCAANGTCAN
GGTCTCCTCTTTTTTTTTTTTTTTCACTGTCTATCTTTGAACCTGTTATCGTCTTCCCGCCAAGGCTATGT
GTCTCCGCCTTTTTTATTTTTTTTACGCCGTTGATTTTCCGCGTTCGTCTCCAACCCCAGACCGACCTCGT
GTGTATGTTGGCTCCTCCCACTCCCCGGGGCGTCTA

Supplementary Figure S1. Sequence of internal chromosome-HHV6 junctions from two CI-HHV6 carriers. (**A**) The sequence from the CI-HHV-6A carrier 3-10q26.3 (24) was obtained using the inverse PCR method (25). The 1.3kb amplicon generated comprised a short subtelomeric sequence (blue text) followed by a region containing telomere (TTAGGG, black text) and degenerate repeats (red text) followed by pure $(TTAGGG)_n$ that could be derived from the human telomere or the viral DR_R-T2 region. This is followed by sequence from the viral genome (brown italic text). (**B**) The sequence from the HGDP1065 CI-HHV-6B carrier was obtained from the 1.6kb amplicon generated using the SubT17-539 primer that anneals to some human subtelomeric sequences and the DR8F primer (see Fig. 2). The sequences are coloured, underlined and in italics as in panel a. Notably all the junction fragments isolated contain regions with degenerate repeats such as TTGGGG (red, underlined) and TGAGGG (red italic) indicating that the virus integrated into the proximal region of an existing telomere that includes degenerate repeats.

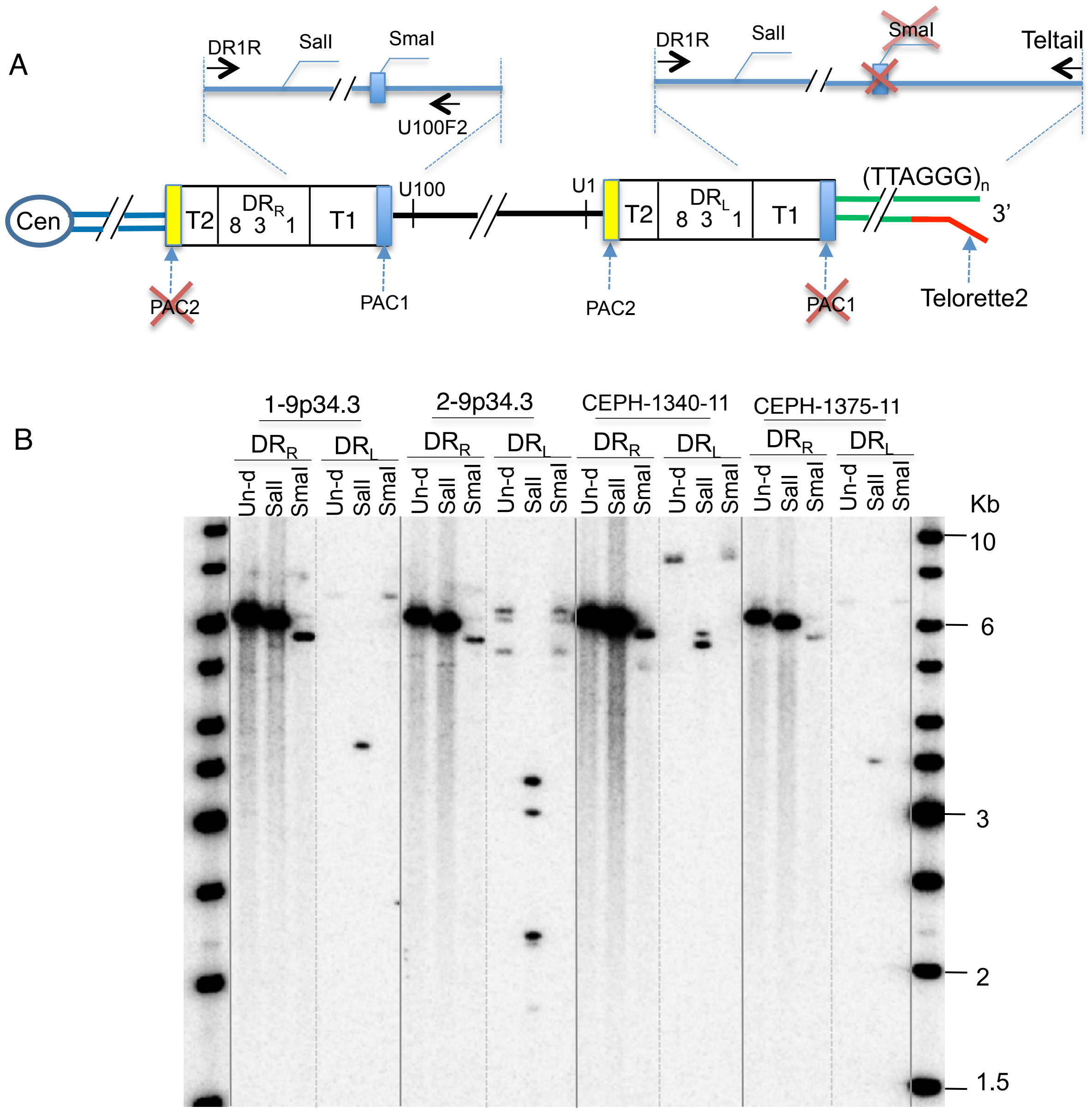

Supplementary Figure S2. Confirmation that the PAC1 sequence is present in DR$_R$ but absent from the terminal DR$_L$ in CI-HHV-6B carriers. (**A**) The diagram explains the assay used to detect the PAC1 sequence that contains a *Sma*I restriction site. (**B**) The PCR amplicon from the internal DR$_R$ (generated with the DR1R and U100F2 primers) in four unrelated CI-HHV-6B carriers is shown undigested (Un-d); digested with *Sal*I (positive control for digestion); digested with *Sma*I to detect the PAC1 sequence. These amplicons were cleaved by *Sma*I in all four donors showing that PAC1 is present in DR$_R$. STELA products were generated from DR$_L$ in the four CI-HHV-6B carriers (1 to 3 amplicons per donor). The STELA products are shown undigested, digested with *Sal*I or *Sma*I. All the amplicons are cleaved by *Sal*I but none were cleaved by *Sma*I showing that the PAC1 sequence is missing from DR$_L$ at the distal end of the integrated viral genome. The products were detected by Southern blot hybridization to a ^{32}P labeled (TTAGGG)$_n$ probe.

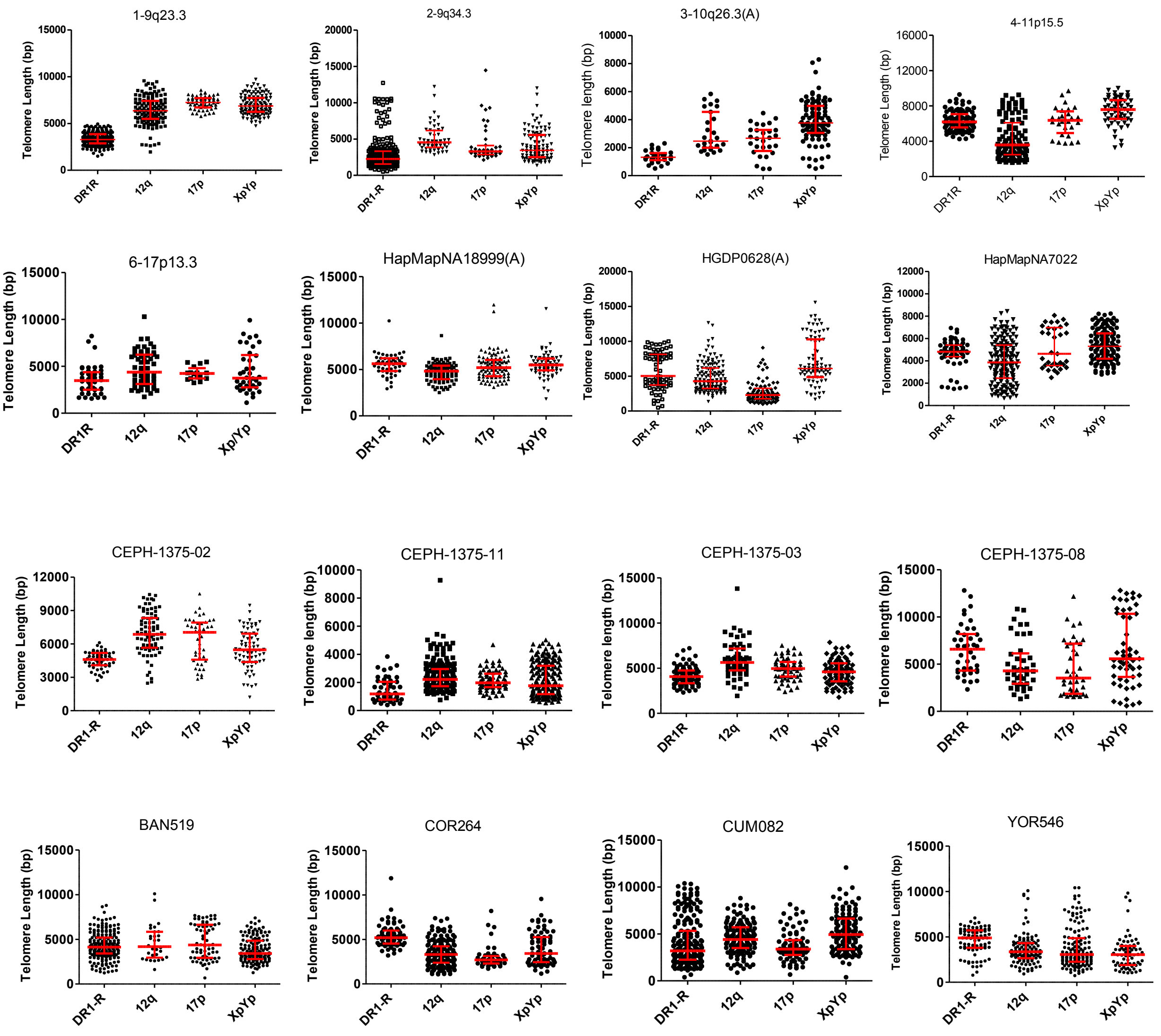

Supplementary Figure S3A (LCLs)

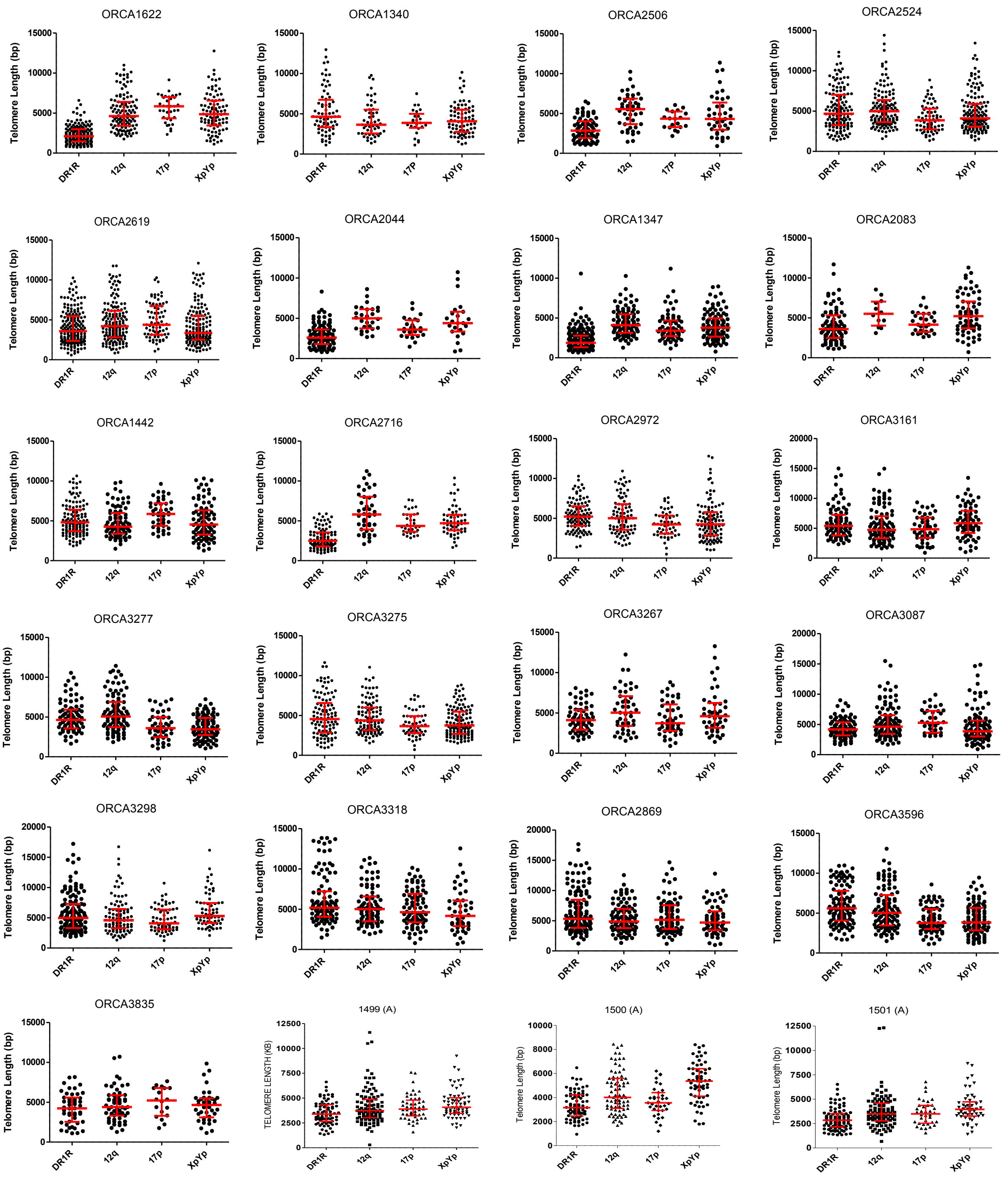

Supplementary Figure S3B (Blood DNAs)

Supplementary Figure S3. Comparative length analysis of the HHV-6 associated, 12q, 17p and XpYp telomeres in 40 CI-HHV-6A or B carriers. (**A**) The scatter plots derived from STELA length analysis at the four telomeres in 16 DNA samples derived from LCLs from CI-HHV-6 carriers. Samples 3-10q26.3, HapMapNA18999 and HGDP0628 are from CI-HHV-6A and the remainder from CI-HHV-6B carriers. (**B**) The scatter plots derived from STELA length analysis at the four telomeres in 24 DNA samples derived from blood from CI-HHV-6 carriers. Samples 1499, 1500 and 1501 are from CI-HHV-6A and the remainder from CI-HHV-6B carriers. The median value and inter-quartile range are shown as red lines.

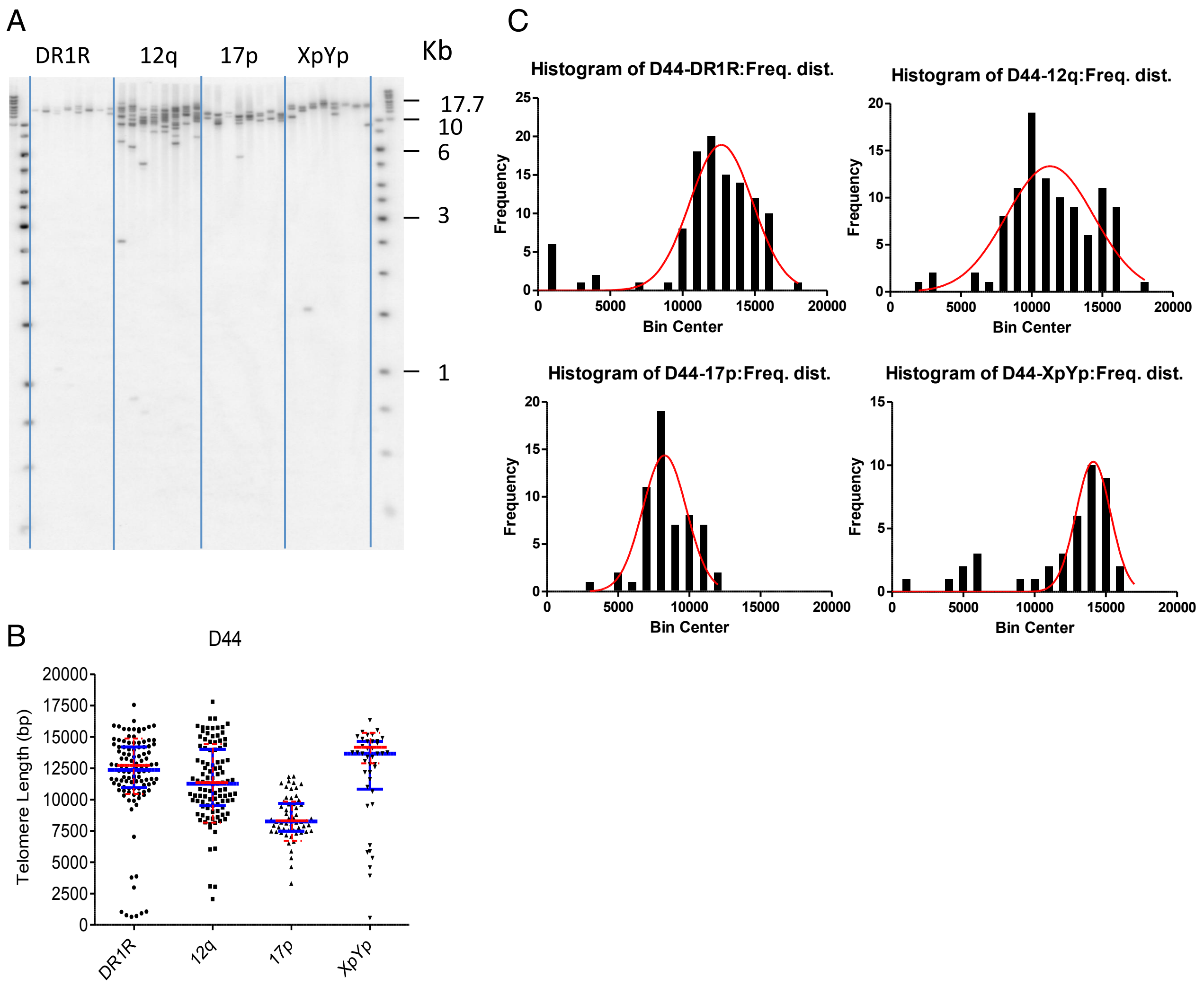

Supplementary Figure S4. Telomere length analysis in sperm DNA. (**A**) A representative STELA blot used to measure telomere length in sperm DNA from donor 44 (D44). The blot shows amplicons from the CI-HHV-6-associated (DR1R), 12q, 17p and XpYp telomeres. (**B**) Summary scatter plots from the telomere length analysis in sperm DNA from D44. The median length and inter-quartile range are shown as blue lines and the mean from the best-fit normal distribution (shown in **c**) and standard deviation (SD) are shown as red lines. (**C**) The sperm telomere molecules were assigned to 1kb bins based on their length and the frequency of molecules in each bin plotted against length. A best-fit normal curve of the population of larger molecules was used to determine the mean length and SD.

A

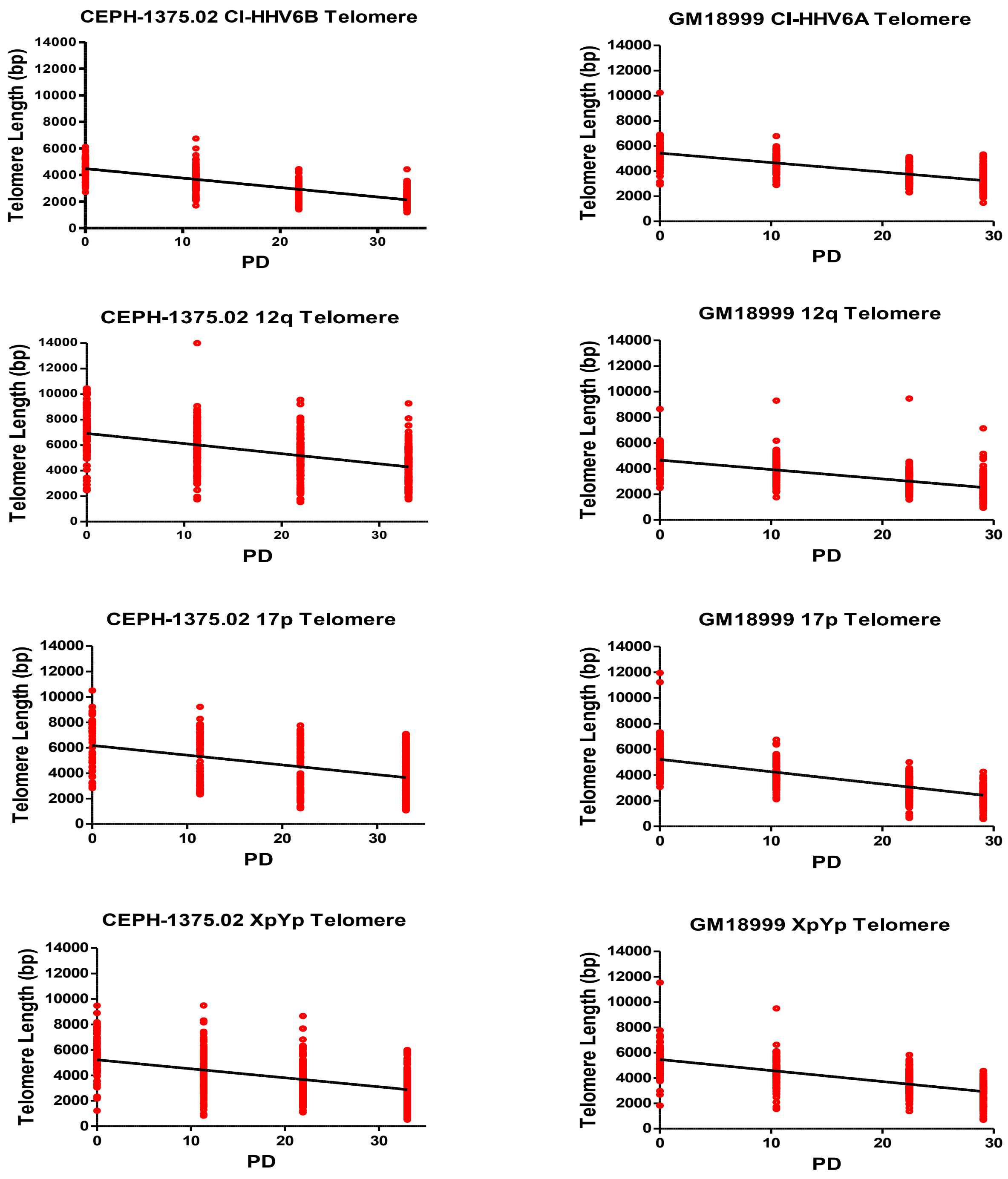

B

Telomere	Telomere shortening rates in CEPH1375.02 LCL	Telomere shortening rates in GM18999 LCL
CI-HHV6 telomere	71 ± 4	75 ± 5
12q telomere	80 ± 7	73 ± 4
17p telomere	77 ± 8	96 ± 4
XpYp telomere	71 ± 6	87 ± 5

Supplementary Figure S5. The rate of telomere shortening in LCLs from a CI-HHV-6A and a CI-HHV-6B carrier. (**A**) Telomere length (bp) plotted against increasing number of population doublings (PD) for the CI-HHV-6-associated, 12q, 17p and XpYp telomeres in the LCLs from CEPH1375.02 CI-HHV-6B and HapMapNA18999 CI-HHV-6A (GM18999). Linear regression analysis was used to produce a line of best-fit. (**B**) Table showing the telomere shortening rate (bp/PD) ± standard error at the four telomeres in each cell line.

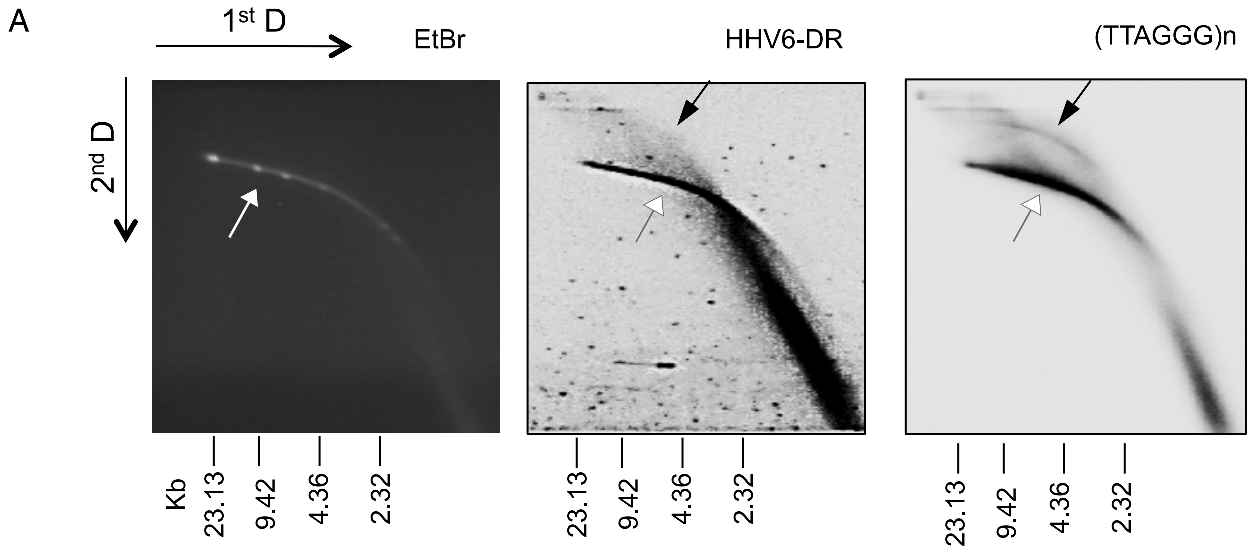

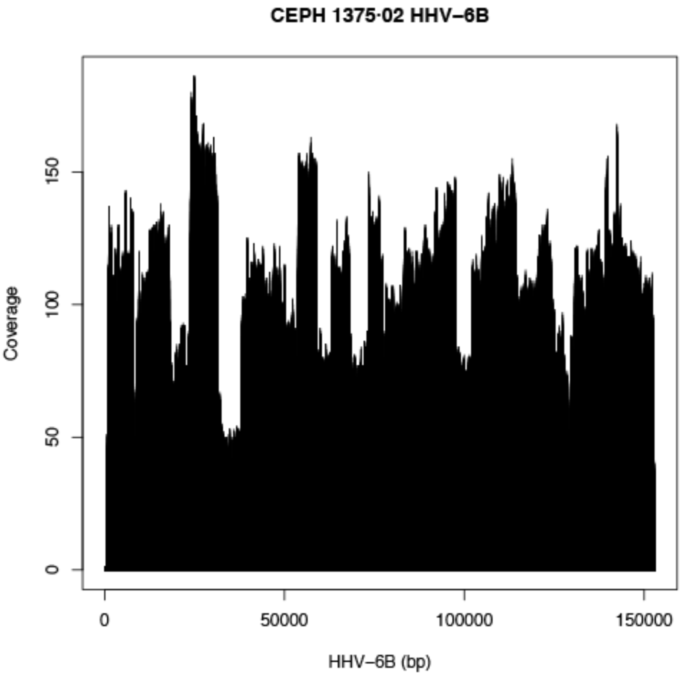

Differences between HHV-6B HST and CI-HHV-6B in CEPH1375.02:

282 single base changes – none predicted to truncate ORFs

Indels: 10 single base insertions; 2 x 2 base insertions; 1 x 3 base insertion

6 single base deletions

None of the indels are predicted to cause frame-shifts. For example the U36 ORF includes 4 base substitutions, 2 x single base insertions and 2 x two base insertions (net gain 6 bases) relative to the HST strain. The predicted HHV-6B U36 amino acid sequence in CEPH1375.02 is 482aa and shows 99.8% identify to the Z29-HHV-6B U36aa sequence.

Homopolymer tracts: we found the IonTorrent sequence analysis was accurate up to A_{10} or T_{10} and up to C_6 or G_6. Therefore for polyA/T tracts >9 and polyC/G >5 we assumed the sequence in 1375-CI-HHV-6B was the same as the reference sequence HST.

Two segments, over the repetitive R3 region and the U1 region are currently unfinished.

Supplementary Figure S6. Extra-chromosomal circular DNA containing DR sequences and summary of sequence analysis from CI-HHV-6B in CEPH1375.02 (**A**) Two-dimensional agarose gel electrophoresis of genomic DNA from CEPH1375.02 CI-HHV-6B following digestion with *Eco*RI and Plasmid-safe DNase. The panel on the left shows the migration of the linear DNA size marker (sizes shown below); the middle panel shows the 2D gel following Southern blot hybridization to detect HHV-6 DR sequences; the right panel shows the same Southern blot hybridized to the telomere probe. The black arrows shows the arc of the relaxed circular molecules containing DR sequences (middle panel) or $(TTAGGG)_n$ (right panel; these could be derived from all telomeres and are more abundant than the DR containing circles). The white arrows show the migration of residual linear DNA (co-incident with the size marker). **(B)** Diagram showing next-generation sequence coverage of the CI-HHV-6B genome from CEPH1375.02. The short reads generated by IonTorrent sequencing (total = 194,439) included duplicates that were identified and removed using Picard (Sourceforge). Following identification and removal of duplicate reads the plot of coverage (number of reads per base) of the remaining 120,117 reads across DR_L and the unique region was generated using Bedtools. The differences between the CI-HHV-6B in CEPH1375.02 and the HST strain are summarized.
